# PKC Inhibits Sec61 Translocon-Mediated Sarcoplasmic Reticulum Ca^2+^ Leak in Smooth Muscle Cells

**DOI:** 10.3389/fphys.2022.925023

**Published:** 2022-06-28

**Authors:** Adan Dagnino-Acosta, Agustín Guerrero-Hernandez

**Affiliations:** ^1^ Centro Universitario de Investigaciones Biomédicas, CONACYT-Universidad de Colima, Colima, Mexico; ^2^ Departamento de Bioquímica, Centro de Investigación y de Estudios Avanzados, Mexico City, Mexico

**Keywords:** translocon, PKC, sarcoplasmic reticulum, IP3, smooth muscle, SERCA, calcium leak

## Abstract

PKC inhibitors stimulate Ca^2+^ release from internal stores in diverse cell types. Our data indicate that this action cannot be explained by an increased agonist-induced IP_3_ production or an overloaded SR Ca^2+^ pool in smooth muscle cells from guinea pig urinary bladder. The incubation of these cells with three different PKC inhibitors, such as Go6976, Go6983, and BIM 1, resulted in a higher SR Ca^2+^ leak revealed by inhibition of the SERCA pump with thapsigargin. This SR Ca^2+^ leakage was sensitive to protein translocation inhibitors such as emetine and anisomycin. Since this increased SR Ca^2+^ leak did not result in a depleted SR Ca^2+^ store, we have inferred there was a compensatory increase in SERCA pump activity, resulting in a higher steady-state. This new steady-state increased the frequency of Spontaneous Transient Outward Currents (STOCs), which reflect the activation of high conductance, Ca^2+^-sensitive potassium channels in response to RyR-mediated Ca^2+^ sparks. This increased STOC frequency triggered by PKC inhibition was restored to normal by inhibiting translocon-mediated Ca^2+^ leak with emetine. These results suggest a critical role of PKC-mediated translocon phosphorylation in regulating SR Ca^2+^ steady-state, which, in turn, alters SR Ca^2+^ releasing activity.

## Introduction

A transient elevation of cytoplasmic Ca^2+^ ([Ca^2+^]_i_) induces changes in cell behavior such as muscle contraction, secretion, and neurotransmission, among others (Berridge et al., 2003). It is known that chemical, electrical and physical stimuli generate calcium influx at the plasma membrane and Ca^2+^ release from internal stores, producing an increase in the [Ca^2+^]_i_, which results in the activation of specific cellular mechanisms. Since the Ca^2+^ ion is a second messenger with a broad-spectrum role in cell signaling, the Ca^2+^ information is coded in the amplitude, location, and frequency of Ca^2+^ signals (Dolmetsch et al., 1997) to generate different behaviors in the same cell. The main internal calcium store is located in the endoplasmic reticulum (ER) or sarcoplasmic reticulum (SR) in muscle cells. This internal Ca^2+^ store is refilled by the action of Sarco/endoplasmic reticulum Ca^2+^ ATPase (SERCA), which in turn hydrolyzes one ATP and accumulates two Ca^2+^ ions in the ER/SR. This action results in a chemical gradient that provides a driving force for Ca^2+^ discharge upon activation of Ca^2+^ release channels (Toyoshima and Nomura, 2002). In smooth muscle cells, the two main Ca^2+^ release channels are the 1,4,5 inositol trisphosphate receptor (IP_3_R) and ryanodine receptor (RyR) (McGeown, 2004; Sanders, 2008). The leak Ca^2+^ channel is the third element involved in determining the amount of Ca^2+^ contained in the SR. The relevance of leak channels arises from establishing the ER/SR steady-state luminal [Ca^2+^] together with the SERCA pump (Gómez-Viquez et al., 2005). These changes in steady-state imply the existence of SERCA pump reserve to cope with conditions associated with increased ER/SR Ca^2+^ leak. Accordingly, this new steady-state level will increase the cellular energy burden. However, this futile cycle cannot be seen as a waste of energy since it participates in cell events such as thermoregulation (Bal and Periasamy, 2020). Despite the importance that Ca^2+^ leak channels play in establishing the luminal SR Ca^2+^ level, the molecular nature of this leak channel has not been identified yet in smooth muscle. A small fraction of RyRs is open in resting condition serving as Ca^2+^ leak channels (Gómez-Viquez et al., 2003; Dagnino-Acosta and Guerrero-Hernández, 2009). In other cells, ER Ca^2+^ leak channels appear to be different from IP_3_Rs and RyRs, being the translocon one of those ER Ca^2+^ leak channels (Camello et al., 2002; Lomax et al., 2002). Translocon is a pore complex that allows peptide movement in and out of the ER (Rapoport et al., 2017). This channel has a large pore to accommodate proteins, which, unregulated, will rapidly dissipate the ER/SR Ca^2+^ gradient. Indeed, a small peptide coded within the translocon functions as a plug for this channel (Itskanov et al., 2021), reducing its Ca^2+^ leakiness; additionally, bound ribosomes decrease Ca^2+^ release *via* translocon (Crowley et al., 1994; Roy and Wonderlin, 2003; van Coppenolle et al., 2004). Moreover, calmodulin and BiP proteins also inhibit the Ca^2+^ leak activity of translocon (Erdmann et al., 2011; Schäuble et al., 2012).

The question is whether the translocon normally functions as ER/SR leak channel (Lemos et al., 2021), particularly in smooth muscle cells (Amer et al., 2009). Translocon in immortalized cell lines appears to be part of the ER Ca^2+^ leak (Flourakis et al., 2006; Schäuble et al., 2012; Linxweiler et al., 2017). However, freshly isolated smooth muscle cells do not present an open translocon, based on the observation that SERCA pump inhibition does not reduce luminal SR Ca^2+^ levels even after several minutes (Gómez-Viquez et al., 2003, 2005).

In smooth muscle cells, Ca^2+^ release from the SR is triggered by IP_3_-producing agonists and by conditions that activate RyRs (Dagnino-Acosta and Guerrero-Hernández, 2009). Localized activation of clusters of RyRs produces Ca^2+^ sparks in smooth muscle cells, those located near the plasma membrane activate a cluster of Ca^2+^-dependent K^+^ channels generating what is known as spontaneous transient outward currents (STOCs) (Muñoz et al., 1998). Agonists that activate PLC via G proteins induce the hydrolysis of PIP_2_ phospholipid in stoichiometric amounts of IP_3_ and DAG, while the former activates IP_3_Rs causing the transient elevation of the [Ca^2+^]_i_; the latter activates PKC and phosphorylates different proteins. Active PKC decreases Ca^2+^ release by phosphorylating membrane receptors (Ishida et al., 2021) or PLCβ (Yue et al., 2000), resulting in a substantial reduction in the agonist-mediated IP_3_ production and, in turn, Ca^2+^ release.

We have studied the mechanism used by inhibitors of PKC to facilitate agonist-mediated Ca^2+^ release in smooth muscle cells, particularly Go6976 because this inhibitor stimulates Ca^2+^ release while inhibiting the carbachol-induced IP_3_ response. This apparent paradox was clarified by showing that PKC inhibitors trigger SR Ca^2+^ leak that seems to be occurring via translocon, and SERCA pumps compensate for this leak. This new steady-state in the luminal SR Ca^2+^ level facilitates agonist-induced Ca^2+^ release. Our results suggest that PKC activity might be modulating translocon-mediated SR Ca^2+^ leak, which modifies SR Ca^2+^ releasing activity.

## Materials and Methods

### Ethics Statement on Animal Use

All animal care and experimental procedures were performed according to the Mexican Official Norm for the Use and Care of Laboratory Animals (NOM-062-ZOO-1999). The protocol was reviewed and approved by the local Ethics Committee on Animal Experimentation (CICUAL, Cinvestav) with 0306–06 and renewed with the reference number 0131–15. Animals were bred and housed in the Cinvestav Animal facility with clean air and controlled light and temperature. Food and water were given *ad libitum*. Precautions were implemented to minimize animal use and reduce pain and distress.

### Isolation of Single Smooth Muscle Cells From Guinea Pig Urinary Bladder

Single smooth muscle cells were isolated using the previously described method (Dagnino-Acosta and Guerrero-Hernández, 2009). The urinary bladder was surgically removed from male guinea pigs, followed by urothelium detachment. The cleaned detrusor muscle (200 mg) was minced in 20 mg pieces; these pieces were digested with preactivated collagenase and papain. Contaminating DNA was minimized with two cycles of incubation with DNAse I for 15 min each in dissection solution. Single smooth muscle cells were obtained by gentle mechanical dispersion with a plastic pipette, and the quantity and quality of cells for each preparation were verified with an optical microscope. Cells were then resuspended in fresh dissection solution and loaded for 1 h in the dark with Mag-Fluo4/AM, followed by two centrifugations at 500 x g with new recording saline solution, and the cell pellet was resuspended in recording saline solution. Smooth muscle cells were stored at 4°C for a minimum of 2 hours before being used the same day.

### Calcium Measurements in Intact Smooth Muscle Cells Loaded With Fura-2/AM

Smooth muscle cells were loaded with 2 μM Fura-2/AM in the dark for 1 h at room temperature. Fura-2 loaded smooth muscle cells were placed on the stage of an inverted microscope and dually excited with a PTI DeltaRamV attached to a Nikon Diaphot TMD inverted microscope. Fluorescence excited at 340 and 380 nm were recorded at 510 nm in response to the addition of 20 mM caffeine, 100 μM carbachol, or 10 μM thapsigargin dissolved in recording saline solution and applied using a puffer pipette placed 10 μm away from the cell. These compounds were applied to the cell with a WPI PV830 pico pump (Aguilar-Maldonado et al., 2003).

### Simultaneous Recording of Both the Cytoplasmic (Fura-2) and the SR Luminal [Ca^2+^] (Mag-fluo4) in Single Patch-Clamped Dialyzed Smooth Muscle Cells

Smooth muscle cells were loaded with 5 μM Mag-Fluo4/AM in the dark at room temperature for 1 h. Mag-Fluo4 loaded smooth muscle cells were placed on the stage of an inverted microscope connected to a PTI microfluorometer as described before. Single smooth muscle cells were patch clamped in the whole-cell configuration, with a holding membrane potential of 0 mV, and dialyzed with a pipette solution containing 100 μM Fura-2 free acid for at least 10 min to ensure that Mag-Fluo4 was removed from the cytoplasm and at the same time loading cells with enough Fura-2 to reliably measure changes in the [Ca^2+^]_i_ (Dagnino-Acosta and Guerrero-Hernández, 2009).

### Spontaneous Transient Outward Currents (STOCs) Recording With the Patch-Clamp Technique

To record spontaneous transient outward currents (STOCs), single muscle cells were patched in the whole-cell configuration and held at 0 mV. The frequency of STOCs was determined for 1 min. Digitized ion current data was stored to be analyzed offline. The frequency of STOCs was determined using the peak detector tool of the Origin program using a threshold ≥20 pA (Gómez-Viquez et al., 2003).

### Quantification of Inositol 1,4,5-Trisphosphate Production (IP_3_) with the Mass Assay

To determine IP_3_ production, we followed a procedure previously described (Rueda et al., 2002b). Briefly, the cerebella of 12 male rats were homogenized in a solution containing Tris-HCl (50 mM), EDTA (1 mM), and mercaptoethanol (1 mM) at pH 8.0 using 15 ml of solution per gr of tissue. Residual tissue debris and nuclei were eliminated by centrifugation at 20,000 x g for 15 min. The resulting pellet was resuspended using a ratio of 2.5 mg of protein per ml of solution, and the IP_3_R-enriched microsomes were kept at -20°C for 2 days before being used for IP_3_ binding assays. Competitive binding between *myo*-[^3^H]inositol 1,4,5-trisphosphate radioactive and nonradioactive IP_3_ gave us an IC_50_ of approximately 10 nM with a Hill coefficient close to 1. The nonspecific binding was obtained using 4 µM of nonradioactive IP_3_.

The amount of 1,4,5-trisphosphate IP_3_ produced in 25 mg of smooth muscle detrusor preincubated in Li solution with or without PKC inhibitors and thapsigargin for 30 min was determined with and without 100 μM carbachol stimulation for 5 s. The IP_3_ production was stopped with 24% perchloric acid solution and kept at four°C. The IP_3_ containing solution was adjusted to a pH 8 with a solution containing TRIS (100 mM), EDTA (50 mM), and KOH (1.5 M). The amount of IP_3_ produced was calculated using the fraction of labeled IP_3_ released from cerebellar IP_3_R microsomes with the equation: IP_3_ = k (1-b)/b, where the k value was obtained for each determination using 75 and 150 pmol of IP_3_ standards. The b value was obtained with the equation b = (sample-NS)/(Bo-NS), where Bo was one of the standards, and NS was the nonspecific binding signal (Rueda et al., 2002b).

### Solutions and Chemicals

The dissociation solution contained (in mM): 55 NaCl, 6 KCl, 5 MgCl_2_, 10 glucose, 80 NaOH, 80 glutamic acid, and 10 HEPES, pH 7.4 (NaOH). The recording saline solution contained (in mM): 137 NaCl, 5 KCl, 4 NaHCO_3_, 2 CaCl_2_, 2 MgCl_2_, 0.42 KH_2_PO_4_, 10 glucose, and 10 HEPES, pH 7.4 (NaOH). The pipette internal solution contained (in mM): 80 glutamic acid, 80 KOH, 5 NaCl, 40 KCl, 2 MgCl_2_, 2 Na_2_ATP, 0.1 GTP, 20 HEPES, pH 7.2 (KOH). The internal solution also contained 100 μM of Fura-2 acid in experiments where SR and cytosolic calcium were measured simultaneously. The Li solution contained (in mM): 120 NaCl, 10 LiCl, 4 KCl, 2 NaHCO_3_, 2 CaCl_2_, 1 MgSO_4_, 10 glucose, and 10 HEPES, pH 7.4 (NaOH).

Mag-Fluo4/AM, Fura-2/AM, and Fura-2-free acid were purchased from Molecular Probes (Eugene, OR). Thapsigargin was purchased from RBI. All other chemicals were from Sigma-Aldrich. Fura-2/AM, Mag-Fluo4/AM, Go6976, Go6983, BIM1, and Thapsigargin were dissolved in a DMSO 1000x stock solution and stored before use. Fura-2-free acid and carbachol were dissolved in water, and aliquots were stored separately at -20°C before being used. Daily freshly dissolved caffeine was prepared in the recording saline solution. All experiments were performed at room temperature (24°C).

### Data and Statistical Analysis

Data for IP_3_ [Ca^2+^]_i,_ luminal SR Ca^2+^ level, and STOCs are reported as mean ± SEM, where n represents either the number of cells studied or the number of independent experiments to determine IP_3_ production. Statistical analysis was carried out with Student`s *t*-test or ANOVA using Dunnett or SNK posthoc analysis. The differences were considered significant when *p* < 0.05.

## Results

### Inhibition of PKC With Go6976 Inhibits Carbachol-Induced IP_3_ Response

**FIGURE 1:**
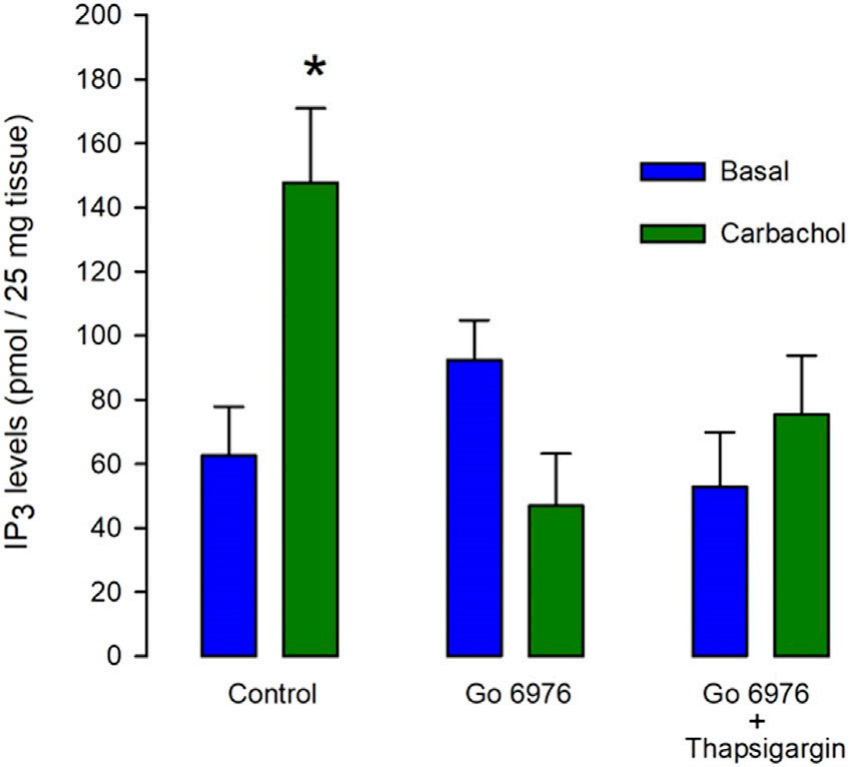
The carbachol-induced IP_3_ production is decreased by Go6976, a specific inhibitor of PKC. Guinea pig urinary bladder smooth muscle fragments were incubated with carbachol (100 μM) for 5 s (left green bar) or vehicle (left blue bar), and IP_3_ levels were measured using the mass assay. Different tissue fragments were preincubated with Go6976 (200 nM) for 30 min (central bars) or with the combination of Go6976 (200 nM) and thapsigargin (100 nM) before the addition of carbachol (right bars). IP_3_ levels are expressed as average pmols produced in 25 mg of tissue ±standard error, *n* = 6. ANOVA, significance was determined with Newman_Keuls post-hoc test with *p = 0.0041.

In smooth muscle cells from guinea pig urinary bladder, the interaction of carbachol with muscarinic receptors leads to PLCβ activation, resulting in the production of both inositol 1,4,5 trisphosphate (IP_3_) and diacylglycerol (DAG). The incubation with carbachol (100 μM) for only 5 s significantly increased more than double the IP_3_ production (Figure 1, left green bar). It is known that PMA-activated PKC inhibits IP_3_ production in smooth muscle (Brock et al., 1985; Aguilar-Maldonado et al., 2003). It is plausible then that incubation with inhibitors of PKC might increase IP_3_ production. If the PKC-feedback loop is absent in this cell type, these inhibitors should not affect agonist-induced IP_3_ production. Unexpectedly, the preincubation of muscle tissue with 200 nM Go6976, a potent inhibitor of classic isoforms of PKC (Gordge and Jonathan Ryves, 1994), for 30 min, totally inhibited the carbachol-induced IP_3_ response (Figure 1, middle green bar. *n* = 6). Although the presence of lithium should be inhibiting 1IP phosphatase in our assay conditions (Hallcher and Sherman, 1980), which is one of the IP_3_ degradation pathways, it is feasible that the agonist-induced Ca^2+^ increase might stimulate a Ca^2+^-dependent IP_3_ kinase limiting the rise in IP_3_ levels. Therefore, we incubated smooth muscle with 100 nM thapsigargin (to deplete SR Ca^2+^ stores) and 200 nM Go6976 for 30 min before the stimulation of IP_3_ production with carbachol (Figure 1, right bars). However, the inhibition of the [Ca^2+^]_i_ response with thapsigargin did not restore the agonist-induced IP_3_ production. This analysis suggests that Go6976 strongly inhibits IP_3_ production.

### The Incubation With Go6976 Potentiates Agonist-Induced [Ca^2+^]_i_ Responses

**FIGURE 2:**
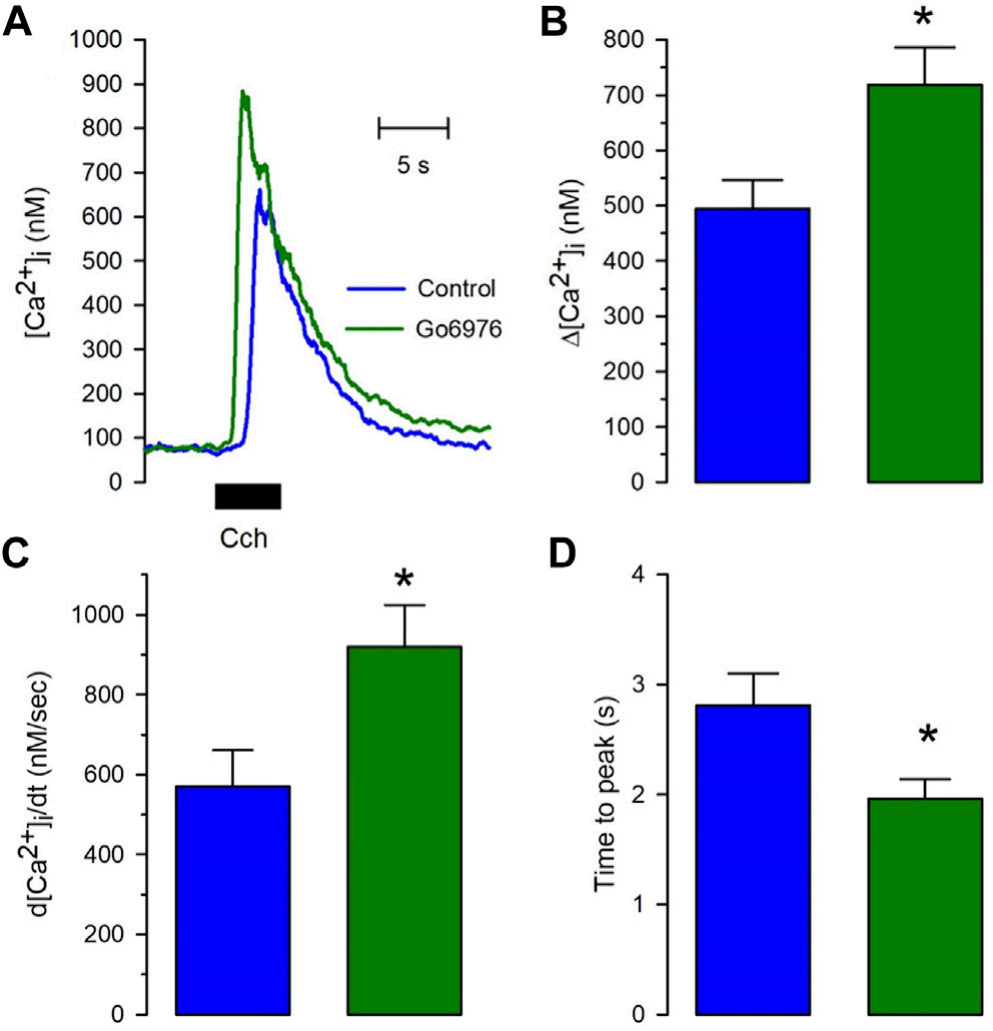
The calcium release induced by carbachol is potentiated in cells pretreated with Go6976. Single smooth muscle cells were loaded with Fura-2/AM and incubated with or without 200 nM Go6976 for 30 min before the exposure to 100 μM carbachol for 5 s using a puffer pipette placed next to the cell. **(A)** Representative carbachol-induced [Ca^2+^]_i_ response in cells incubated with (green trace) and without Go6976 (blue trace). **(B)** The peak amplitude with (green bar) and without Go6976 (blue trace). **(C)** The peak rate of [Ca^2+^]_i_ rise with (green bar) and without Go6976 (blue trace). **(D)** Time to the peak of the carbachol-induced [Ca^2+^]_i_ response with (green bar) and without Go6976 (blue bar). The bars show the average ±standard error for control (*n* = 11) and Go6976 (*n* = 19). Significance was assessed using Student´s t-test. *p < 0.05.

**FIGURE 3:**
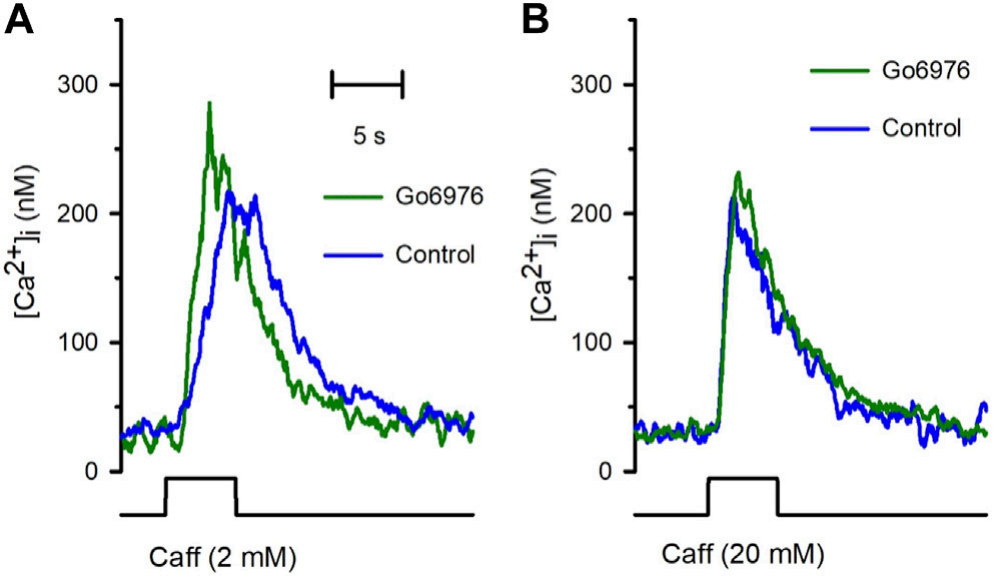
The incubation with Go6976 potentiates caffeine-induced [Ca^2+^]_i_ responses. Single smooth muscle cells loaded with Fura-2/AM were stimulated with caffeine using a puffer pipette placed next to the cell for the time indicated in the bottom black trace (5 s). **(A)** Representative traces of the [Ca^2+^]_i_ response for cells preincubated with (green trace) and without (blue trace) Go6976 and stimulated with 2 mM caffeine at the time indicated (bottom black trace). **(B)** Representative traces of the [Ca^2+^]_i_ responses stimulated with 20 mM of caffeine for cells preincubated with (green trace) and without (blue trace) Go6976 (*n* = 8).

Despite the strong reduction in the carbachol-induced IP_3_ response, the carbachol-induced [Ca^2+^]_i_ response was significantly elevated (Figure 2, green trace vs. blue trace). All the parameters examined were significantly modified by the incubation with 200 nM Go6978. Indeed, the amplitude of the carbachol-induced [Ca^2+^]_i_ response (Figure 2B) went from 494 ± 52 nM (*n* = 11) to 718 ± 67 nM (*n* = 19), the peak rate of rise (Figure 2C) increased from 570 ± 91 nM/s (*n* = 11) to 919 ± 105 nM/s (*n* = 19) while the rising time (Figure 2D) was significantly decreased since it went from 2.81 ± 0.29 s (*n* = 11) to 1.96 ± 0.18 s (*n* = 19) by the inhibition of PKC with Go6976. Since Go6976 did not promote a larger carbachol-induced IP_3_ production, we studied the possibility that the incubation with Go6976 could result in an overloaded SR Ca^2+^ store. The smooth muscle SR is endowed with the IP_3_ receptor and Ryanodine Receptor (RyR); these receptors share the same SR Ca^2+^ store in this cell type (Rueda et al., 2002a). The application of low levels of caffeine (2 mM) displayed a faster and increased [Ca^2+^]_i_ response (Figure 3A, solid line) that went from 149 ± 7 nM (*n* = 8, blue trace) to 233 ± 25 nM (*n* = 8, green trace) in cells incubated with 200 nM Go6976. A saturating concentration of caffeine (20 mM) that activates all RyR at once can be used as an indirect indicator of the size of the SR Ca^2+^ store. In this case, caffeine-induced [Ca^2+^]_i_ response did not display any difference between cells incubated with Go6976 (Figure 3B, green trace, 211 ± 26 nM, *n* = 8) and control (Figure 3B, blue trace, 166 ± 8 nM, *n* = 8). These data show that inhibition of PKC potentiates both the carbachol- and caffeine-induced [Ca^2+^]_i_ responses, but it seems unlikely that this was due to an overloaded SR Ca^2+^ store.

### The Inhibition of PKC Increases the Ca^2+^ Leak From the SR Store

**FIGURE 4:**
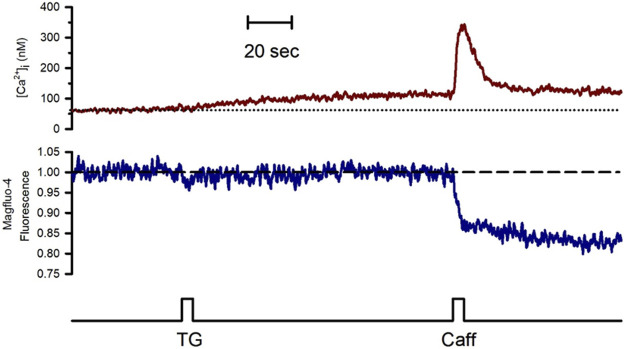
The inhibition of the SERCA pump with thapsigargin revealed a weak SR Ca^2+^ leak in the freshly isolated guinea pig urinary bladder. Single smooth muscle cells previously loaded with Mag-fluo-4/AM were dialyzed with the whole configuration of the patch-clamp technique (to remove the excess of cytoplasmic Mag-fluo-4) with a pipette solution containing Fura-2 free acid and kept at 0 mV to record the [Ca^2+^]_i_ (red trace) and the luminal SR Ca^2+^ level (blue trace). The application of 10 μM thapsigargin with a puffer pipette placed next to the cell at the time indicated by the bottom black trace resulted in a slow and slight elevation of the [Ca^2+^]_i_ with no reduction in the luminal SR Ca^2+^ level. Representative trace of *n* = 10.

**FIGURE 5:**
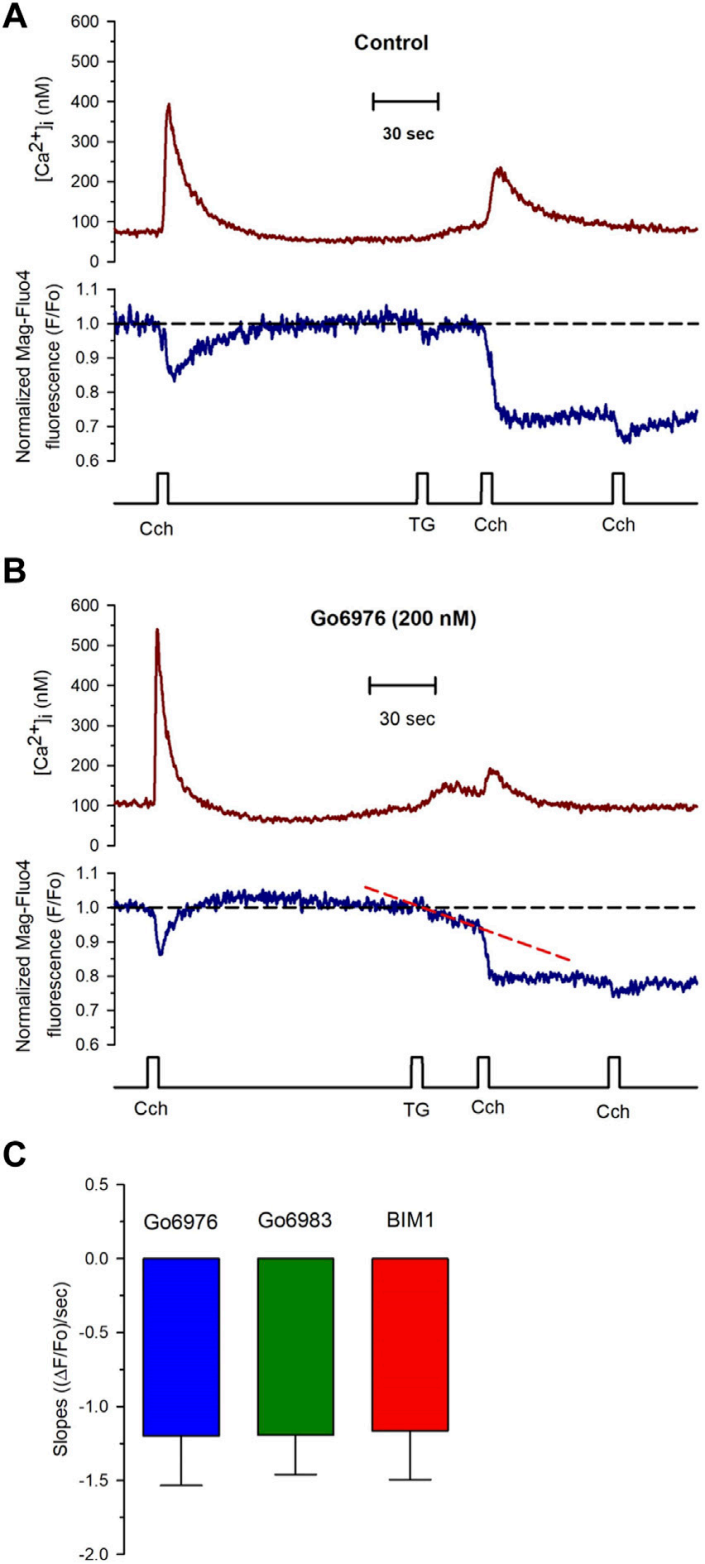
PKC inhibitors generate a compensated SR calcium leak in smooth muscle cells revealed with the application of thapsigargin. Single smooth muscle cells previously loaded with Mag-fluo-4/AM were dialyzed with the whole configuration of the patch-clamp technique (to remove the excess of cytoplasmic Mag-fluo-4) with a pipette solution containing Fura-2 free acid and kept at 0 mV to record the [Ca^2+^]_i_ (red trace) and the luminal SR Ca^2+^ level (blue trace). **(A)** Representative traces of control cells exposed to 100 μM carbachol at the time indicated, followed by the exposure to 10 μM thapsigargin and another application of carbachol 30 s after thapsigargin. The application of thapsigargin did not reduce the luminal SR Ca^2+^ level. **(B)** Representative traces were obtained from smooth muscle cells incubated with Go6976. The application of thapsigargin revealed an apparent SR Ca^2+^ leak, as indicated by the red dashed line. **(C)** Smooth muscle cells were incubated with different PKC inhibitors such as Go6976 (blue bars, *n* = 8), Go6983 (green bar, *n* = 6), and BIM1 (red bar, *n* = 7), and the thapsigargin-induced SR Ca^2+^ leak slope was determined as the mean ± error standard for the number of indicated cells.

To study the effect of Go6976-induced PKC inhibition on the luminal SR Ca^2+^ level, we simultaneously recorded changes in Mag-fluo4 and Fura-2 fluorescence to measure modifications in the luminal SR (Figure 4, blue trace) and the [Ca^2+^]_i_ (Figure 4, brown trace), respectively. We have previously shown that the SR Ca^2+^ store does not present an evident Ca^2+^ leak under our recording conditions (Gómez-Viquez et al., 2003, 2005; Dagnino-Acosta and Guerrero-Hernández, 2009). Indeed, applying 10 μM thapsigargin for 5 s to inhibit the SERCA pump resulted in a small increase in the [Ca^2+^]_i_ because this pump no longer buffers the plasma membrane Ca^2+^ entry (Figure 4, dotted line). Still, there was no discernible SR Ca^2+^ leak for the recorded time (Figure 4, dashed line). We have previously shown that inhibition of SERCA pumps produces a large reduction in the Ca^2+^ availability to release channels in smooth muscle cells (Gómez-Viquez et al., 2003; Dagnino-Acosta and Guerrero-Hernández, 2009). Accordingly, the agonist-induced [Ca^2+^]_i_ response was significantly diminished (Figure 5A, second application of carbachol) since the [Ca^2+^]_i_ response was 159 ± 15 nM (*n* = 7). However, the same protocol but in cells incubated with 200 nM Go6976 resulted in a clear reduction in the SR Ca^2+^ level in response to the inhibition of SERCA pumps with thapsigargin (Figure 5B, red dashed line). This effect was associated with a transient elevation of the [Ca^2+^]_i_ suggesting an increased SR Ca^2+^ leak and a faster reduction of the SR Ca^2+^ content upon thapsigargin application. Indeed, a second stimulation with carbachol induced a significantly smaller [Ca^2+^]_i_ response (78 ± 17 nM, *n* = 5). The third application of carbachol-induced no further reduction of the luminal SR Ca^2+^ level nor any increase in the [Ca^2+^]_i_ (Figure 5). These observations support the contention that the 5-s application of 10 μM thapsigargin irreversibly inhibited SERCA pumps. Moreover, although the luminal SR Ca^2+^ level was not calibrated as an absolute value of [Ca^2+^]_SR_, the luminal SR Ca^2+^ nadir (-0.06 ± 0.02 ΔF/Fo, *n* = 7) was not different (-0.07 ± 0.02 DF/Fo, *n* = 5) when cells were incubated with 200 nM Go6976. If Go6976 had induced a Ca^2+^ overloaded SR, a smaller Ca^2+^ leak should be expected since a less leaky SR would be easier to overload with Ca^2+^. Nevertheless, Go6976 increased the SR Ca^2+^ leak.

Go6976 inhibits classic PKCs and PKD (DAVIES et al., 2000). To determine whether PKD was participating in the process, we used Go6983, another inhibitor of PKC that does not inhibit PKD. Incubation of cells with 200 nM of Go6983 induced an SR Ca^2+^ leak similar to the one caused by Go6976 (Figure 5C, green bar). These data suggest that inhibition of PKCs was responsible for the induction of the SR Ca^2+^ leak. Indeed, incubation of smooth muscle cells with 5 μM BIM 1 also produced the same magnitude of SR Ca^2+^ leak based on the slope of SR Ca^2+^ reduction (Figure 5C, red bar). It has been reported that Go6976 increases the activity of EGF receptors (Shah et al., 2005). We decided to incubate cells with Go6976 and AG1478, an inhibitor of the EGF receptor (Shah et al., 2005). The slope of the thapsigargin-induced reduction in the luminal SR Ca^2+^ level (1.17 × 10^−3^ ± 0.34 × 10^−3^ ((ΔF/Fo)/sec), *n* = 4) was similar to the one obtained with only Go6976. These data imply that the effect of Go6976 was due to the inhibition of PKCs.

### Inhibition of PKC Increases SR Ca^2+^ Leak via the Translocon in Smooth Muscle Cells

**FIGURE 6:**
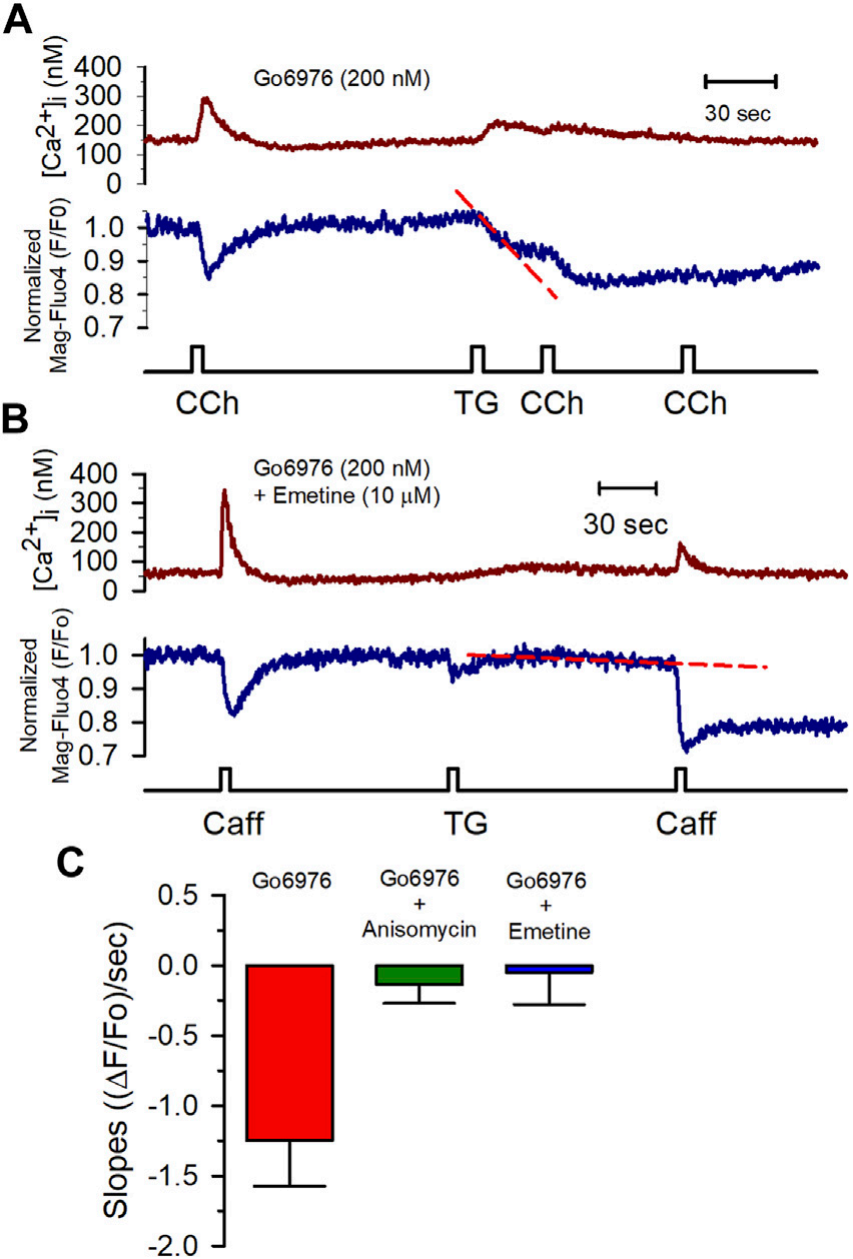
Anisomycin and emetine blocked the Go6976-induced SR Ca^2+^ leak. **(A)** [Ca^2+^]_i_ (red trace) and [Ca^2+^]_SR_ (blue trace) changes in response to CCh (100 μM) from a smooth muscle cell incubated with Go6976 (200 nM). The application of thapsigargin resulted in a rapid and partial depletion of the [Ca^2+^]_SR_ (red dashed line) with strong inhibition of the CCh-induced responses. **(B)** Caffeine-induced changes in the [Ca^2+^]_i_ (red trace) and the [Ca^2+^]_SR_ (blue trace) from a smooth muscle cell incubated with both Go6976 (200 nM) and emetine (10 μM). The application of TG resulted in a much smaller SR Ca^2+^ leak (red dashed line). **(C)** The slope of the SR signal obtained after blocking the SERCA pump with thapsigargin was evaluated in cells incubated with Go6976 and vehicle (red bar, *n* = 5), 200 μM anisomycin (green bar, *n* = 4), and 10 μM emetine (blue bar, *n* = 6) and plotted as the average ±standard error. Dunnett post-hoc with *p < 0.05.

**FIGURE 7:**
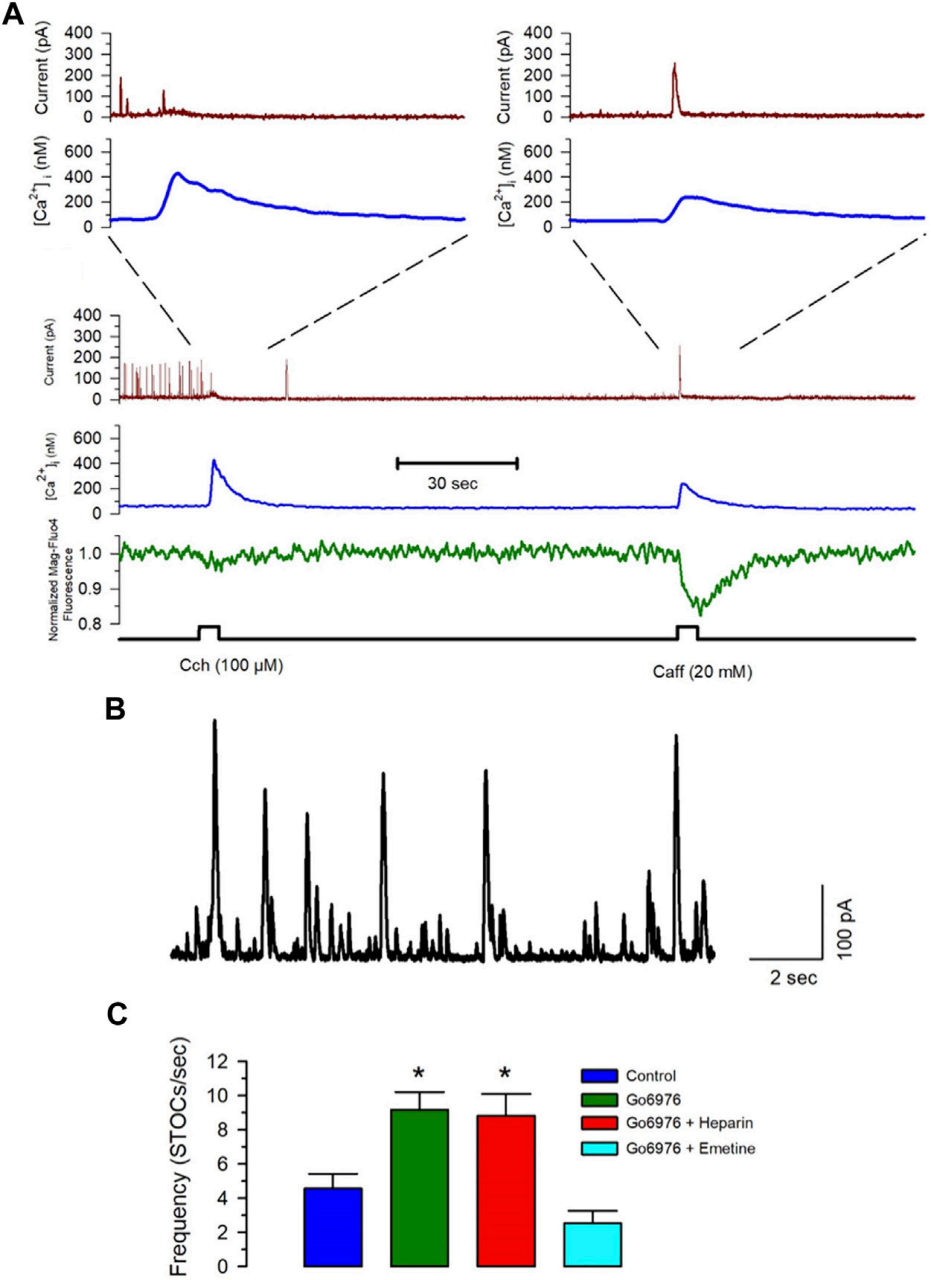
Emetine blocks the Go6976-induced increased STOC frequency. Freshly isolated smooth muscle cells were loaded with Mag-fluo-4/AM and dialyzed with Fura-2 free acid in the pipette solution while holding membrane potential at 0 mV. **(A)** The spontaneous and calcium-sensitive potassium currents were recorded (brown trace) together with the [Ca^2+^]_i_ (blue trace) and the luminal SR Ca^2+^ level (green trace) during the application of carbachol or caffeine at the time indicated (bottom black trace). Both stimuli induced a significant elevation of the [Ca^2+^]_i,_ with the main difference that carbachol. However, inhibited STOCs, did not cause a generalized activation of STOCs, while caffeine clearly stimulated transient outward currents. **(B)** Representative trace of STOCs in a control smooth muscle cell. **(C)** Frequency of STOCs per second plotted as the average ±standard error for control (blue bar), with 200 nM Go6976 (green bar), with 200 nM Go6976 with 5 mg/ml heparin in the pipette solution (red bar), and with 200 nM Go6976 and 10 μM emetine (cyan bar). ANOVA, Dunnett post-hoc test, *p < 0.05.

All these data show that PKC inhibition activated an SR Ca^2+^ leak, implying the activation of release channels (IP_3_Rs or RyRs) or some other type of Ca^2+^-permeable channel in the SR. 2 mM MgCl_2_ inside the pipette solution inhibits the RyR-mediated SR Ca^2+^ leak. Yet, this condition did not interfere with the Go6976-induced SR Ca^2+^ leak (Figures 5B, C). Additionally, the IP_3_Rs do not appear to participate in the enhanced SR Ca^2+^ leak based on the observation that the presence of heparin in the pipette solution did not decrease the enhanced STOCs frequency induced by Go6976 (Figure 7C). Translocon is a pore that allows the nascent peptide to reach the ER lumen while functioning as a Ca^2+^ leak in the ER (van Coppenolle et al., 2004; Linxweiler et al., 2017). We decided to study whether PKC inhibition might be activating the translocon. We used two previously reported translocon-mediated Ca^2+^ leak inhibitors, emetine and anisomycin. They inhibit protein translation by stalling the nascent peptide in the ribosome (van Coppenolle et al., 2004; Ong et al., 2007; Amer et al., 2009). The presence of 10 μM emetine inhibited the effect of 200 nM Go6976 on the stimulation of Ca^2+^ leak (Figures 6A,B). Emetine has decreased the leakiness of the SR Ca^2+^ store, so the second stimulation displayed a larger [Ca^2+^]_i_ response than with only Go6976 vs. Figures 6A,B. The presence of anisomycin (200 μM, Figure 6C) or emetine (10 μM, Figure 6C) significantly reduced the slope of thapsigargin-induced SR Ca^2+^ leak (compare the slopes of the red dashed lines in vs. Figures 6A,B).

### A New Steady State in the SR Ca^2+^ Content Stimulates RyR-Mediated Ca^2+^ Release

Spontaneous transient outward currents (STOCs) are potassium currents carried out by Ca^2+^-dependent large-conductance K^+^ channels activated by a Ca^2+^ spark due to the coordinated opening of a small cluster of RyRs (ZhuGe et al., 1999; Cheranov and Jaggar, 2002). Figure 7A shows that Ca^2+^ release by IP_3_R (carbachol application) did not increase the BKCa current, while caffeine activated the BKCa current with the same increase in the [Ca^2+^]_i_. The frequency of STOCs increases with a higher RyR activity (ZhuGe et al., 1999; Cheranov and Jaggar, 2002). The incubation of cells with 200 nM Go6976 significantly increased the frequency of STOCs (Figure 7C, green bar, *n* = 5), suggesting that the inhibition of PKC increased the spontaneous activation of RyRs. Moreover, the inhibition of IP_3_Rs with 5 mg/ml of heparin in the pipette solution did not modify the effect of Go6976 (Figure 7C, red bar, *n* = 5). However, the presence of emetine (10 μM) fully inhibited the Go6976-induced increased STOCs frequency (Figure 7C, cyan bar, *n* = 6) without interfering with the STOCs amplitude. Collectively, these data suggest that the inhibition of PKC in smooth muscle cells induces a translocon-mediated SR Ca^2+^ leak that appears to be compensated by an increase in the SERCA pump activity. This new steady-state seems to facilitate the spontaneous activation of RyRs.

## Discussion

We have found that PKC inhibition stimulated Ca^2+^ release from the SR in response to carbachol and caffeine in smooth muscle cells isolated from guinea pig urinary bladder. However, we could not find evidence of an overloaded Ca^2+^ store to explain this effect. Moreover, PKC inhibition reveals a translocon-mediated SR Ca^2+^ leak without reducing the agonist-induced [Ca^2+^]_i_ responses. We have inferred that this situation reflects increased compensatory SERCA pump activity. This new steady-state facilitates Ca^2+^ release by both IP_3_Rs and RyRs. The latter resulted in a higher STOCs frequency. Overall, these data point to the SR Ca^2+^ leak role in determining the steady-state luminal SR Ca^2+^ level, particularly the participation of translocon in defining the SR Ca^2+^ leakage.

### The Translocon Plays a Role in the SR Ca^2+^ Leak in Smooth Muscle Cells

The inhibition of PKC with different inhibitors such as Go6976, Go6983, and BIM1 resulted in a reduction of the luminal SR Ca^2+^ level upon inhibition of the SERCA pump. This SR Ca^2+^ leak was not inhibited by MgCl_2_ or heparin in the pipette solution, arguing against RyRs or IP_3_Rs being the SR Ca^2+^ leak channels responding to the inhibitors of PKC. However, emetine and anisomycin inhibited this SR Ca^2+^ leak supporting the idea that Sec61 translocon participates in this SR Ca^2+^ leak.

The translocon from eukaryotic cells is a protein complex formed by the three subunits of SEC61 (α, β, γ), Sec62, and Sec63 proteins, oligosaccharyltransferase complex (ribophorin I, ribophorin II, and OST48), TRAM, and the TRAP protein complex (TRAP α, β, γ, and δ) (Potter and Nicchitta, 2002). Puromycin, an inhibitor of the protein synthesis that releases the nascent peptides leaving the ribosome attached to the open conformation of the translocon, has been critical for demonstrating the participation of translocon as an ER Ca^2+^ leak in different cell types (Camello et al., 2002; Lomax et al., 2002; Giunti et al., 2007; Ong et al., 2007). However, results obtained in proliferating vascular smooth muscle cells show that translocon could work as an SR Ca^2+^ leak since it is activated by puromycin and inhibited with anisomycin and emetine. Still, it does not participate in the thapsigargin-revealed SR Ca^2+^ leak (Amer et al., 2009). A result we have corroborated here as well. Our data suggest that constitutive PKC activity inhibits smooth muscle translocon SR Ca^2+^ leak activity. However, this effect of PKC is not exclusive to smooth muscle cells since we have data that PKC is also blocking the HeLa cell translocon Ca^2+^ leak activity.

The translocon contains a large pore of 50 Å in diameter, making it difficult for the SERCA pump to avoid Ca^2+^ pool depletion. Accordingly, an extensive account of molecular mechanisms reduces translocon Ca^2+^ leak activity (Lang et al., 2017). The pore ring of SEC61α involves hydrophobic amino acid residues that block the central pore (Voorhees and Hegde, 2016; Itskanov et al., 2021). The plug peptide, calmodulin, and BiP block the translocon Ca^2+^ leak activity (Erdmann et al., 2011; Schäuble et al., 2012; Lang et al., 2017; Rapoport et al., 2017). Mass spectrometry and biochemical studies have shown that several proteins of the translocon complex are phosphorylated in the absence of any stimulation. This phosphorylation is present in SEC61α, β, and γ, Sec62, Sec63, the two subunits of TRAM and the five subunits of TRAP (Gruss et al., 1999; Villén et al., 2007; Daub et al., 2008; Dephoure et al., 2008; Oppermann et al., 2009). Interestingly, SEC61β phosphorylation significantly improves peptide translation efficiency to the luminal part of the SR (Gruss et al., 1999), suggesting a better coupling of translocon and ribosomes. Additional target candidates for PKC phosphorylation under basal activity cannot be excluded (i.e., phosphorylation of the signal recognition particle and its receptor). However, we do not know how and which phosphorylated subunit or related protein reduces translocon Ca^2+^ leak activity.

Although staurosporine is a generalized kinase inhibitor, paradoxically, it can also activate kinases. This is the case, particularly with p38 MAPK (Xiao et al., 1999; Yamaki et al., 2010; Ramiro-Cortés et al., 2011). Moreover, both emetine (Kim et al., 2015) and anisomycin (Xiong et al., 2006; Sampieri et al., 2008) activate p38 MAPK. Additionally, the effect of protein synthesis inhibitors on P38 MAPK activity requires hours of incubation, while the Ca^2+^ leak inhibition occurs much faster. Since these two protein synthesis inhibitors blocked staurosporine-induced ER Ca^2+^ leak and all three inhibitors have the same effect on p38 MAPK, we think it is unlikely that modifications in p38 MAPK activity explain the effect of staurosporine seen in the present study.

### Role of the SR Ca^2+^ Leak in Establishing the New Steady-State

A constant [Ca^2+^]_SR_ results from the balance between Ca^2+^ leak and SERCA pump activity (Berridge et al., 2003; Friel and Chiel, 2008). One way to assess the size of this Ca^2+^ leakage is the fast inhibition of the SERCA pump with thapsigargin and to determine the rate of luminal SR Ca^2+^ reduction. Under our recording conditions, thapsigargin did not produce any rapid decrease in the [Ca^2+^]_SR_. This result cannot be explained by thapsigargin partially inhibiting the SERCA pump. These data indicate a comparable small SR Ca^2+^ leak and a small SERCA pump activity. However, PKC inhibitors increased translocon-mediated SR Ca^2+^ leak, implying that translocon phosphorylation reduces its Ca^2+^ leak activity in resting conditions. Because the PKC inhibitors did not deplete the SR Ca^2+^ pool, there must be a compensatory increase in SERCA pump activity and, arguably, higher ATP consumption.

### Role of SERCA Pump Activity in the Facilitation of Ca^2+^ Release

Currently, the role of SERCA pumps in refilling the ER/SR Ca^2+^ pool is clearly established. However, data suggest that the SERCA pump sensitizes the propagating Ca^2+^ wave-front in secretory cells, facilitating an efficient calcium release (Huang et al., 2006). This conclusion was reached by observing that SERCA pump inhibition reduces the Ca^2+^ wave velocity without affecting the [Ca^2+^]_i_ amplitude or decreasing the luminal calcium content (Keller et al., 2007). Moreover, RGS2 knockout stimulates agonist-induced Ca^2+^ release without evidence of an increased agonist-induced IP_3_ production or higher sensitivity of IP_3_Rs (Wang et al., 2004, 2005).

We have previously shown that SERCA pumps potentiate Ca^2+^ release in smooth muscle cells by a mechanism independent of its SR Ca^2+^ refilling activity (Gómez-Viquez et al., 2003). Blocking the SERCA pump produced a decreased amplitude and velocity of Ca^2+^ release with IP_3_R in HeLa cells (Aguilar-Maldonado et al., 2003) or with IP_3_Rs and RyRs in smooth muscle cells (Dagnino-Acosta and Guerrero-Hernández, 2009). The activation of β adrenergic receptors in heart cells stimulates Ca^2+^ release by increasing the activity of SERCA pumps without any evidence of an overloaded SR Ca^2+^ pool (Zhou et al., 1999; Maxwell and Blatter, 2012). This occurs because there is a compensatory higher RyR2 leak activity (Reinhardt et al., 2021; Nolla-Colomer et al., 2022). Therefore, examples abound showing SERCA pump increased activity facilitates Ca^2+^ release by generating a new steady-state.

### The Role of Leak Channels in Establishing the Steady-State [Ca^2+^] and Their Effect on the SR Physiology

The evidence indicates that translocon presents significant participation in the ER Ca^2+^ leak in pancreatic acinar cells (Lomax et al., 2002), human salivary glands (Ong et al., 2007), liver microsomes from rats (Giunti et al., 2007), and LNCaP cells (van Coppenolle et al., 2004), suggesting that Ca^2+^ leak activity of Sec61α has a critical role in establishing the steady-state ER Ca^2+^ level. However, translocon does not seem to be operating as an SR Ca^2+^ leak channel under basal conditions in smooth muscle cells. Our data suggest that this is due to the constitutive PKC activity that results in a reduced SR Ca^2+^ leak. Nevertheless, not only translocon but other channels function as ER Ca^2+^ leak channels; for instance, the RyRs (Bellinger et al., 2008; Lehnart et al., 2008), the IP_3_Rs (Oakes et al., 2005), TRPV1(Lotteau et al., 2013), Orai2 (Bandara et al., 2013), and Orai3 (Leon-Aparicio et al., 2017), among others. This large variety of ion channels operating as ER/SR Ca^2+^ leak channels argues for the relevance of ER/SR Ca^2+^ leak in cell physiology.

### The SR Functions as an Intelligent Compartment Handling Free Calcium

The calcium release from internal stores is organized in discrete units (Baddeley et al., 2011), resulting in quantal Ca^2+^ release events such as sparks for RyR or puffs for IP_3_Rs (Steenbergen and Fay, 1996; Hajnóczky and Thomas, 1997). The ER/SR is a network of interconnected tubules and cisternae without diffusion barriers; therefore, it is expected that activation of release channels leads to complete depletion of the ER/SR. However, ER/SR shows quantal Ca^2+^ release behavior (Wu and Bers, 2006; McCarron and Olson, 2008). There are several explanations for this phenomenon; one of them is that Ca^2+^ release channels (IP_3_Rs and RyRs) have preferential access to Ca^2+^ trapped in luminal proteins (Guerrero-Hernandez et al., 2010; Guerrero-Hernández et al., 2020), making an efficient Ca^2+^ release event. While the leak channels control SERCA pump activity by modifying the free luminal ER/SR [Ca^2+^], implying much smaller Ca^2+^ fluxes than when the total Ca^2+^ capacity of the ER/SR is involved. This separation between Ca^2+^ release channels and leak channels allows the ER/SR to function as a Ca^2+^ source without triggering ER stress by Ca^2+^ depletion or other activities that require an elevated free luminal [Ca^2+^]_ER_.

Altogether, our results indicate that PKC inhibitors increase the Ca^2+^ leak activity of translocon, which appears to be compensated by a higher SERCA pump activity to avoid SR Ca^2+^ depletion. This increased steady-state results in accelerated Ca^2+^ release in response to activation of either IP_3_Rs or RyRs. These results point to the scenario where the translocon Ca^2+^ leak modulates the Ca^2+^-releasing activity of the SR Ca^2+^ store.

## Data Availability

The original contributions presented in the study are included in the article/supplementary material, further inquiries can be directed to the corresponding author.
